# The Relationship between Training Load Indicators and the Total Quality Recovery Scale during the Preseason Period in Collegiate Taekwondo Athletes

**DOI:** 10.5114/jhk/217225

**Published:** 2026-04-02

**Authors:** Chan-Ho Park, Hyun Chul Jung

**Affiliations:** 1 Department of Physical Education, Graduate School, Kyung Hee University, Yongin, Republic of Korea.; 2 Department of Sports Coaching, Kyung Hee University, Yongin, Republic of Korea.; 3 Sports Science Research Center, Kyung Hee University, Yongin, Republic of Korea.

**Keywords:** training monitoring, recovery status, session rating of perceived exertion, exponentially weighted moving average

## Abstract

This study examined the relationship between training load (TL) indicators and recovery status (total quality recovery, TQR) during the preseason training period in collegiate Taekwondo athletes. A total of 25 athletes (17 males and 8 females; age: 19.2 ± 1.04 years, training experience: 6.8 ± 3.40 years, body height: 168.0 ± 8.08 cm, body mass: 64.1 ± 8.38 kg, fat content: 15.9 ± 7.21%) participated in this prospective observational study. Over eight weeks, 855 training sessions were monitored. TL was quantified using the session rating of perceived exertion (sRPE) method, and derived TL indicators [weekly load variation (SDΔ), rolling average acute:chronic workload ratio (RA ACWR), exponentially weighted moving average acute:chronic workload ratio (EWMA ACWR), training monotony (TM), and training strain (TS)] were calculated. Recovery status was assessed daily using the TQR scale. In the univariate linear mixed model analyses, SDΔ (β = −0.30, 95% CI [−0.51, −0.08], p = 0.006) and the EWMA ACWR (β = −0.46, 95% CI [−0.64, −0.29], p < 0.001) were significantly associated with TQR. However, in the multivariate model, only the EWMA ACWR (β = −0.74, 95% CI [−0.97, −0.52], p < 0.001) remained a significant predictor. These findings suggest that the EWMA ACWR is the most sensitive TL indicator for capturing short-term changes in recovery status. Integrating the EWMA ACWR with simple tools such as the TQR scale may help coaches balance TL and recovery in preseason training. Future studies should include additional physiological and psychological markers to evaluate whether EWMA ACWR-based load management can effectively reduce injury risk and enhance athletic performance.

## Introduction

One of the primary goals of preseason training is to improve physical fitness in preparation for the upcoming in-season (Jeong et al., 2011). Preseason training typically involves higher frequency and intensity, often resulting in two to four times greater training loads (TLs) compared with in-season periods (Jagim et al., 2021; Jeong et al., 2011). They must strike a careful balance between maximizing positive physiological adaptations and minimizing the risks of overtraining, impaired recovery, and injury (Buchheit et al., 2013). Therefore, it is essential to monitor and adjust individual responses to training during the preseason period to ensure effective adaptation and reduce the risk of injury (Borresen and Lambert, 2009; Buchheit et al., 2013; Jagim et al., 2021).

Effective monitoring of TL requires an integrated approach that accounts for physiological, psychological, social, and mechanical factors, with outcomes that can be applied in real-time within training environments (Bourdon et al., 2017; Kellmann et al., 2018). Numerous methods have been proposed to quantify TLs, including both external and internal measures, among which the session rating of perceived exertion (sRPE) is the most widely adopted. The sRPE calculates TLs by multiplying session duration by the perceived exertion rating (CR-10 scale), and this method demonstrated high correlations with heart-based metrics (r = 0.75–0.90) (Foster, 1998; Haddad et al., 2017). While effective in quantifying internal TLs across various sports, excessive TLs without adequate recovery are associated with reduced performance and increased injury risk (Kellmann et al., 2018; Saw et al., 2016). To assess recovery status, various indicators such as performance testing, biological sampling, the heart rate, and subjective self-reporting have been employed (Claudino et al., 2017; Coutts et al., 2007; Nunes et al., 2014; Williams et al., 2017a). Among these, the subjective self-report method has proven more sensitive to changes in TLs than physiological and performance-based markers (Saw et al., 2016; Thorpe et al., 2017). In particular, the total quality recovery (TQR) scale is a valid and practical tool for monitoring athletes' perceived recovery, as it correlates well with TLs and the demands of competition (Debien et al., 2020; Kenttä and Hassmén, 1998; Sansone et al., 2020).

To optimize training prescription, sports scientists have developed indicators that quantify the balance between TL and recovery (Foster, 1998; Hulin et al., 2016; Wu et al., 2024). One such an indicator is training monotony (TM), calculated by dividing the mean TL over the past 7 days by its standard deviation (SD), reflecting the repetitiveness of training patterns (Foster, 1998; Wu et al., 2024). However, TM alone does not capture the total workload. Training strain (TS), calculated by multiplying weekly TL by TM, incorporates both magnitude and variability with high values linked to impaired recovery and increased injury risk (Foster, 1998). The rolling average-based acute:chronic workload ratio (RA ACWR), derived from the Banister’s fitness-fatigue model, compares the acute workload (7 days) to the chronic workload (28 days) to estimate preparedness (Hulin et al., 2016). Despite its popularity, the RA ACWR has been criticized for oversimplifying the complex interaction between fitness and fatigue (Murray et al., 2017; Williams et al., 2017b). To address these limitations, Williams et al. (2017b) proposed the exponentially weighted moving average-based acute:chronic workload (EWMA ACWR) method which applies time-decayed weighting, making it more responsive to sudden workload changes. The EWMA ACWR has demonstrated greater sensitivity than the RA ACWR in predicting non-contact injuries (Murray et al., 2017). More recently, some studies have proposed new TL indicators based on weekly load variation and its SD (Lazarus et al., 2017; Tysoe et al., 2020; Wu et al., 2024). It has been reported that an increase in training loads exceeding 1 SD is associated with an elevated injury risk, with the risk becoming higher when the increase exceeds 2 SD (Lazarus et al., 2017; Tysoe et al., 2020; Wu et al., 2024).

Taekwondo (TKD) is a globally practiced martial art and an Olympic sport consisting of three primary disciplines: sparring, poomsae, and demonstration (Jeong and Chun, 2022; Kazemi et al., 2009). Poomsae is a non-contact discipline in which athletes perform a predetermined sequence of offensive and defensive techniques against an imaginary opponent (Jeong and Chun, 2022; Kazemi et al., 2016). Poomsae involves abrupt stops, rapid directional changes, and high difficulty techniques, making athletes susceptible to overuse injuries (Jeong and Chun, 2022; Kazemi et al., 2016). Previous studies have shown that non-contact and overuse injuries have been identified as primary causes of injury among poomsae athletes (Jeong and Chun, 2022; Kazemi et al., 2016). These overuse injuries are largely attributed to cumulative TLs and insufficient recovery rather than competition itself. We believe that injuries not only impair performance, but may also hinder long-term athletic development.

Despite these concerns, relatively few studies have examined the physiological and psychological responses to TLs in TKD athletes (Jeong and Chun, 2022; Kazemi et al., 2016), and research focusing specifically on poomsae athletes is even more limited. Thus, this study aimed to examine the relationship between TL indicators and recovery status (i.e., TQR) during the preseason training period in collegiate TKD athletes. The study sought to identify which TL indicators would contribute to reduced recovery to provide foundational insights for the future preseason training program design.

## Methods

### Participants

A total of 25 collegiate TKD athletes (male: n = 17, female: n = 8) participated in this study. The descriptive characteristics of the participants are presented in Table 1. All participants were officially registered as collegiate athletes with the Korea TKD Association (KTA). They had achieved awards in nationally recognized, KTA-approved competitions within the past three years. This study was conducted in accordance with the principles of the Declaration of Helsinki. All participants were informed of the purpose, procedures, and potential risks, and provided written consent using a form approved by the Institutional Review Board of the Kyung Hee University, Yongin, Republic of Korea (protocol code: KHGIRB-25-139; approval date: 09 June 2025).

**Table 1:** Descriptive characteristics of the collegiate TKD athletes (n = 25).

Variables	Male (n = 17)	Female (n = 8)	All (n = 25)
Age (years)	19.2 (1.01)	19.3 (1.16)	19.2 (1.04)
Training experience (years)	6.5 (3.26)	7.5 (3.82)	6.8 (3.40)
Body height (cm)	171.7 (6.65)	160.1 (4.14)	168.0 (8.08)
Body mass (kg)	67.7 (6.72)	56.4 (6.18)	64.1 (8.38)
BMI (kg·m^−2^)	23.0 (2.23)	22.0 (2.34)	22.7 (2.27)
Percent fat (%)	12.1 (3.64)	24.0 (6.27)	15.9 (7.21)

Note. The data are the mean and standard deviation. BMI: body mass index

### Design and Procedure

This study employed a prospective observational design. A total of 855 training sessions were monitored over eight weeks. The preseason training program was designed entirely by two team coaches, without interference from the researchers. Each training session included a light warm-up, technical drills, strength and conditioning, and a cool-down phase.

### Data Collection

During the preseason period, TLs for each training session were monitored using the sRPE method. The sRPE was calculated by multiplying the duration of the training session (in minutes) by the RPE score measured using the Borg CR-10 scale, and was expressed in arbitrary units (AU) (Foster et al., 2001). The RPE was self-reported by participants using a paper questionnaire within 15–30 minutes after the end of each training session (Foster et al., 2001). All participants were already familiar with the use of the RPE. The training session duration was defined as the total time from the start of the warm-up to the end of the cool-down. On rest days or when participants did not attend training, the TL for that day was recorded as 0 AU. The collected daily TL data were used to calculate derived variables (i.e., TL indicators).

The TQR scale was used to monitor recovery status (Kenttä and Hassmén, 1998). The TQR scale ranges from 6 to 20 (Kenttä and Hassmén, 1998). Participants recorded their TQR scores each day before the first training session by responding to the question: “How do you feel about your recovery?”. A score of 13 or higher was considered indicative of at least an adequate level of recovery (Kenttä and Hassmén, 1998). To examine the relationship between TLs and recovery, recovery scores recorded on the day following each training session were used for analysis.

### Calculation of Training Load Indicators

The TL indicators were calculated using the sRPE method. All indicators were analyzed using a rolling-based method to account for daily fluctuations, which allowed for continuous daily monitoring of the relationship between TLs and recovery. The TL indicators were calculated as follows: the standard deviation of the change in the workload over the past 7 days (SDΔ) represented the difference between the 7-day workload and the average weekly workload, relative to the variance in the weekly workload (Lazarus et al., 2017; Wu et al., 2024). SDΔ was calculated as follows:


$$SD\Delta =\frac{Last\;7-day\;Workload-Average\;of\;Weekly\;Workload}{Variance\;of\;weekly\;Workload}$$

The RA ACWR was calculated by dividing the acute workload by the chronic workload (Hulin et al., 2016). The acute workload was defined as the sum of the daily workload over the past 7 days (Hulin et al., 2016). The chronic workload was calculated as the average acute workload over the past 4 weeks (Hulin et al., 2016). The RA ACWR was calculated as follows:


$$RA\;ACWR=\frac{Acute\;Workload}{Chronic\;Workload}$$

The EWMA was calculated based on a selected time decay constant (λ) (Williams et al., 2017b). The λ value, calculated as λ = 2/(*N* + 1), ranges between 0 and 1 and represents the decay rate of the load value (Williams et al., 2017b). The value of N was set to reflect acute workload (7 days) and chronic workload (28 days) periods. Thus, the decay constant was set to 0.25 for the acute workload and 0.069 for the chronic workload. The EWMA ACWR was calculated as follows:


$$\begin{array}{l} EWMA_{today}=Workload_{today}\times \lambda +\left(1-\lambda \right)\times EWMA_{yesterday}\\ EWMA\;ACWR=\frac{EWMA\;Acute\;Wokrload}{EWMA\;Chronic\;Workload} \end{array}$$

TM was calculated by dividing the 7-day average workload by the SD of the workload over the 7 days (Foster, 1998). TM was calculated as follows:


$$TM=\frac{Average\;of\;Last\;7-day\;Workload}{Standard\;Deviation\;of\;Last\;7-day\;Workload}$$

TS was calculated by multiplying weekly TM by the workload over the past 7 days (Foster, 1998). TS was calculated as follows:


TS=Sum of Last 7−day Workload×Monotony

### Statistical Analysis

Statistical analyses were performed using R (version 4.4.3, R Foundation for Statistical Computing, Vienna, Austria) with the lme4, lmerTest, and emmeans packages. The TL indicators were converted to within-individual z-scores. To analyze the relationship between TL indicators and recovery status, a linear mixed-effects model (LMM) was employed. Univariate LMM analyses were conducted to explore the individual relationships between each TL indicator and TQR. Subsequently, multivariate LMM analyses were performed to evaluate the independent effects of multiple predictors on recovery status. In the multivariate analysis, multicollinearity was assessed using correlation coefficients and variance inflation factors (VIFs), with the observed VIF value being 1.63. For variables that were significant in the multivariate model, participants were classified into three groups (low, moderate, and high) based on within-individual tertiles. Differences in TQR across these tertiles were then analyzed. When significant main effects were observed within the model, post hoc comparisons were conducted using the Bonferroni correction. To account for repeated measures, athlete ID was included as a random effect in all LMMs. In the univariate and multivariate analyses, estimates (*β*), standard errors (SE), and 95% confidence intervals (CIs) were reported. For group comparison analyses, estimated marginal means (emmean) and 95% CIs were presented. The level of significance was set at α = 0.05.

## Results

**Figure 1:**
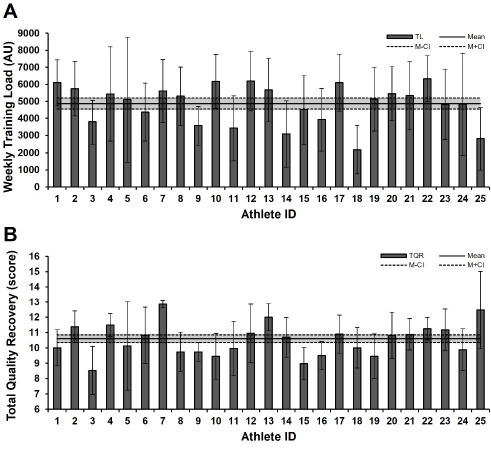
Weekly training load (A) and the weekly TQR score (B) of athletes during the 8-week preseason period. All data are presented as means with 95% confidence intervals (M−CI and M+CI denote the mean minus and plus the 95% confidence interval, respectively). sRPE: session rating of perceived exertion, TL: training load, TQR: total quality recovery, AU: arbitrary units

The average weekly TL during the 8-week preseason period was 4867 AU (95% CI: 4541.8–5192.8), while the average weekly TQR score was 10.6 (95% CI: 10.34–10.86) (Figure 1).

**Figure 2:**
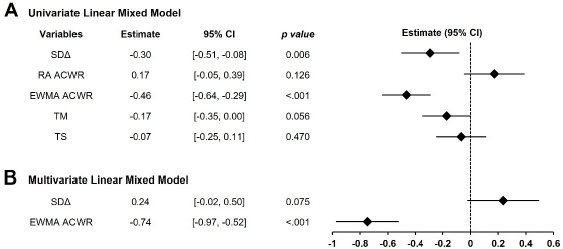
Fixed effect estimates from univariate (A) and multivariate (B) linear mixed model analysis of training load indicators predicting TQR. *95% CI: 95% confidence interval, SDΔ: standard deviation of change, RA ACWR: rolling average-based acute:chronic workload ratio, EWMA ACWR: exponentially weighted moving average-based acute:chronic workload ratio, TM: training monotony, TS: training strain*

**Figure 3:**
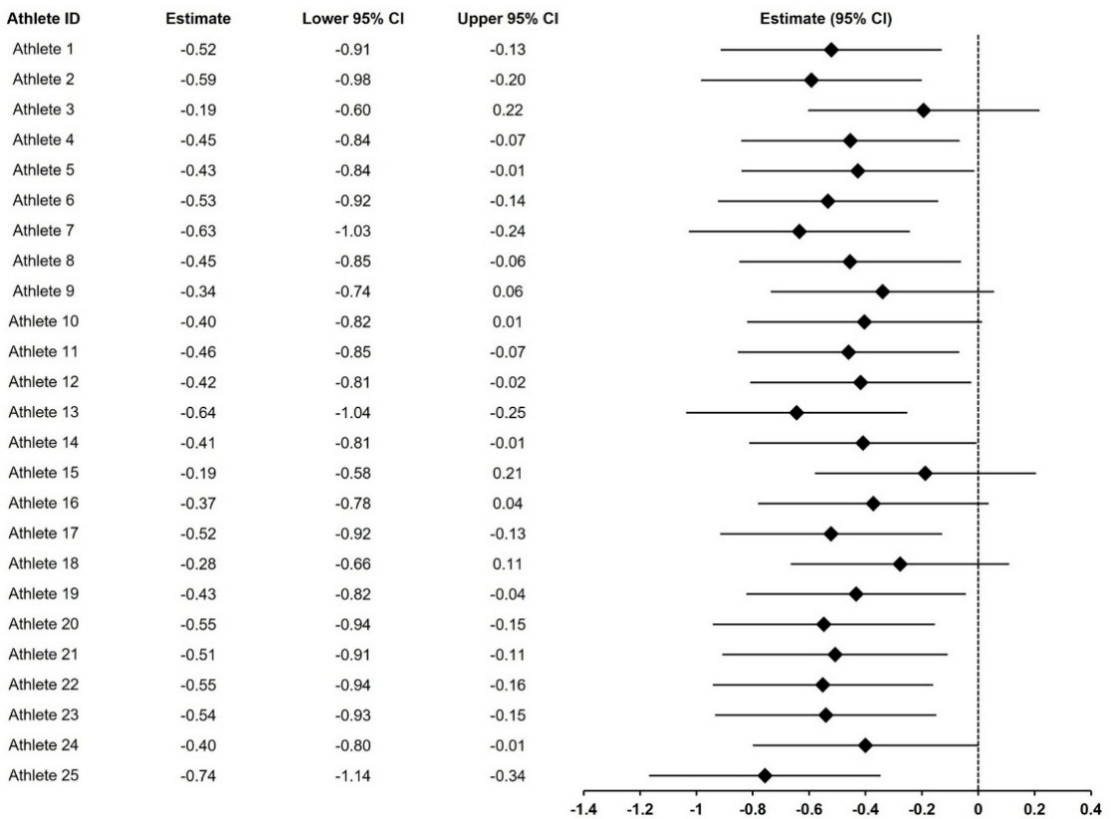
Individuals' EWAM ACWR slopes derived from the univariate linear mixed model. *95% CI: 95% confidence interval*

In the univariate LMM analysis (Figure 2A), among the five derived TL variables, SDΔ (*β* = −0.30, 95% CI [−0.51, −0.08], *p* = 0.006) and the EWMA ACWR (*β* = −0.46, 95% CI [−0.64, −0.29], *p* < 0.001) were significantly associated with TQR. In contrast, the RA ACWR, TM, and TS were not. When SDΔ and the EWMA ACWR were entered into a multivariate LMM (Figure 2B), only the EWMA ACWR remained significant (*β* = −0.74, 95% CI [−0.97, −0.52], *p* < 0.001). At the individual level, 19 of 25 athletes (76%) showed negative slopes in the relationship between the EWMA ACWR and TQR (Figure 3). The remaining six athletes (24%) showed negative slopes, although these were not statistically significant.

**Figure 4:**
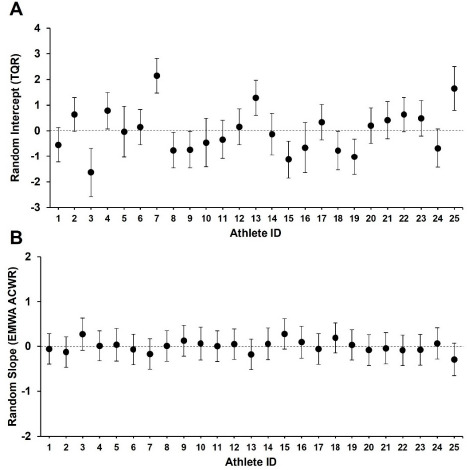
Athlete ID random effects from the linear mixed model analysis of the exponentially weighted moving average predicting total quality recovery. (A) Random intercept of total quality recovery, (B) random slope of the EWMA ACWR. *TQR: total quality recovery, EWMA ACWR: exponentially weighted moving average-based acute:chronic workload ratio*

In the random effect for athlete ID, variability in baseline TQR (intercept) was observed across athletes (variance = 0.95, SD = 0.98). Ten athletes (40%) showed values significantly different from the group mean (Figure 4A). The EWMA ACWR-TQR slopes showed a homogeneous pattern across athletes (variance = 0.05, SD = 0.22), and no athlete differed significantly from the group mean (Figure 4B).

**Figure 5:**
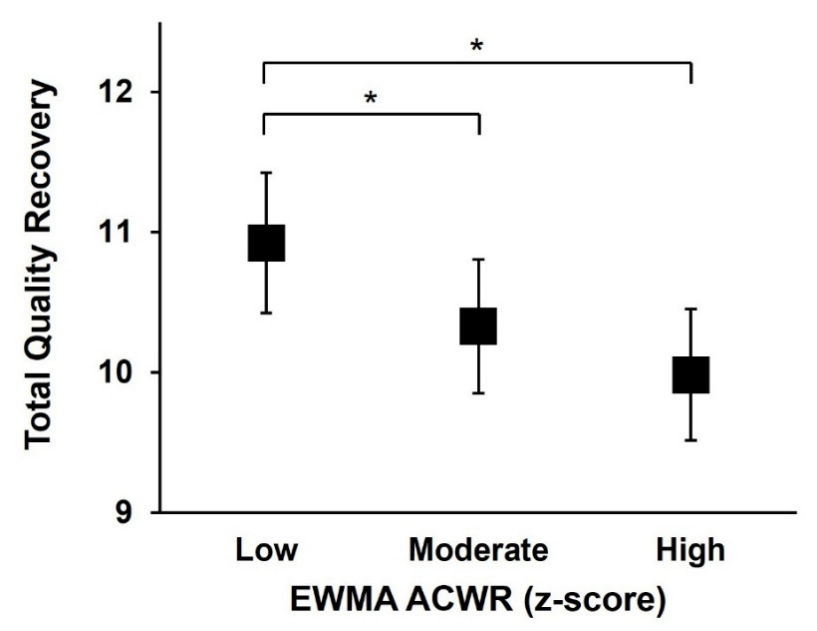
Comparison of estimated marginal means of TQR across EWMA ACWR tertiles (± 95% CI). EWMA ACWR: exponentially weighted moving average-based acute:chronic workload ratio, 95%CI: 95% confidence interval; * indicates significant differences between tertiles (p < 0.05)

When TQR was compared across within-athlete EWMA ACWR tertiles, the highest values were observed in the low EWMA ACWR group (Figure 5). The low EWMA ACWR (emmean = 10.93, 95% CI [10.42, 11.42]) showed significantly higher TQR scores than the moderate EWMA ACWR (emmean = 10.33, 95% CI [9.85, 10.80], *p* = 0.01) and the high EWMA ACWR (emmean = 9.98, 95% CI [9.51, 10.45], *p* < 0.001).

## Discussion

To the best of our knowledge, research analyzing the TL-response relationship in TKD athletes remains limited. This study aimed to identify which TL indicators affected recovery status, to provide foundational insights for designing future preseason training programs. The main finding was that among the five derived TL indicators, only the EWMA ACWR consistently demonstrated a significant negative association with TQR. Although SDΔ was significant in the univariate analysis, the EWMA ACWR remained the only independent predictor in the multivariate model. These results suggest that the EWMA ACWR is the most sensitive indicator of the short-term relationship between TL and recovery status.

Impellizzeri et al. (2020) pointed out the methodological limitations of the ACWR. In fact, Moreno-Pérez et al. (2021) and Seco-Serna et al. (2024) have reported that the RA ACWR represents only one of several factors associated with injury and, by itself, has limited predictive ability. The EWMA ACWR applies greater weighting to recent workloads while also accounting for the cumulative load, making it more sensitive to short-term variations (Williams et al., 2017b). The EWMA ACWR has been reported to demonstrate superior performance in reducing injury risk in sports, such as curling (Wu et al., 2024), football (Murray et al., 2017), and soccer (Tiernan et al., 2022). The finding in this study that the EWMA ACWR is associated with TQR supports previous research results, and the differences observed in the EWMA ACWR tertile analysis further strengthen the consistency of this relationship. Furthermore, these results demonstrate that the EWMA ACWR may serve as an important indicator for injury risk reduction and conditioning strategies during the preseason. A decrease in subjective recovery scores has been reported to be associated with increased cumulative fatigue and injury risk (Timoteo et al., 2021). In addition, although the training volume during an athlete's preseason is high, it is difficult to collect a sufficient sample size for injury occurrence due to the relatively short period. Therefore, monitoring an athlete's recovery status (TQR) through the cumulative load (EWMA ACWR) can be a practical and crucial alternative for mitigating athletes’ fatigue and injury risk.

This study showed a significant negative correlation between SDΔ and TQR in the univariate model, supporting previous research findings that increased weekly TL variability may increase injury risk (Lazarus et al., 2017; Tysoe et al., 2020; Wu et al., 2024). However, in multivariate models including the EWMA ACWR, the effect of SDΔ was not significant in the present study. This suggests that although the VIF between SDΔ and the EWMA ACWR was low (1.63) and there was no severe multicollinearity, the explanatory power of SDΔ was heavily dependent on shared variance with the EWMA ACWR. The EWMA ACWR is an indicator that applies weighted sensitivity to detect abrupt changes in TL while also considering the cumulative load over time (Murray et al., 2017; Williams et al., 2017b). Due to this characteristic, the short-term variability captured by SDΔ can also be incorporated within the EWMA ACWR. In fact, the residual component of SDΔ not explained by the EWMA ACWR did not show a significant relationship with TQR in the multivariate model. In contrast, the EWMA ACWR demonstrated a significant negative association with TQR, even after accounting for its shared variance with SDΔ. This suggests that the EWMA ACWR reflects not only variability but also the influence of temporal weighting and the cumulative load, thereby enabling a more precise and independent evaluation of recovery status. Thus, the EWMA ACWR stands out as a robust and informative metric for monitoring TL and should be prioritized in both the training program design and fatigue management strategies.

In the random effect analysis of the EWMA ACWR-TQR relationship, heterogeneity was observed in the baseline recovery levels (intercept) of individual athletes. Approximately 40% of athletes exhibited baseline recovery levels different from the group average. These results support previous research indicating that an athlete's recovery status is influenced by individual lifestyle factors, such as sleep, nutrition, and stress management (Halson, 2008; Kellmann et al., 2018). However, caution is warranted in this interpretation. TQR used as a recovery indicator in this study is collected based on a subjective questionnaire. Perceived recovery or well-being assessments, such as TQR, do not only reflect pure physiological responses to TL (Kenttä and Hassmén, 1998; Smith, 2003). These indicators can be complexly influenced by various psychosocial confounding factors, such as athletes' academic-life balance, financial burdens, athlete-coach relationships, and family and social stressors (Stallman and Hurst, 2016). Therefore, when interpreting individual differences in baseline recovery levels, this comprehensive context must be considered together. In contrast, the EWMA ACWR-TQR slope, which indicates the degree of the recovery decline with increasing TL, showed little variation among athletes. This suggests that while the absolute recovery level differs among individuals, the tendency for recovery levels to decrease as the EWMA ACWR increases is a universal response consistently observed in athletes. Therefore, athlete management requires maintaining an EWMA ACWR-based load monitoring system applicable to all athletes while implementing customized adjustments that account for each athlete's individual recovery baseline (Li et al., 2025; Patterson et al., 2025).

Kenttä and Hassmén (1998) proposed a TQR score of 13 as the minimum recovery level required for athletes to maintain sustained training. However, the TQR score observed in this study was 10.6, falling below the generally accepted minimum recovery threshold. This suggests that during the preseason period, athletes trained without achieving sufficient recovery. During periods of intensified training, such as the preseason, recovery impairment and fatigue accumulation may become even more pronounced (Kennedy and Drake, 2017; Russell et al., 2022). Kennedy and Drake (2017) reported an increase in neuromuscular fatigue among rugby union players during the latter stages of preseason training. Russell et al. (2022) observed that mental and physical fatigue levels increased as the preseason progressed. Particularly, given that the EWMA ACWR has been shown to have a significant association with recovery impairment, additional TLs imposed on an already low recovery status may further increase the risk of overtraining or injury (Williams et al., 2017a). Williams et al. (2017a) confirmed that when the EWMA ACWR was elevated and recovery was reduced (decreased HRV), the risk of overuse injury increased. Such recovery impairment and fatigue accumulation can become even more problematic when combined with the specific demands of a sport. For example, TKD poomsae is an aesthetic sport where athletes are evaluated not only on physical performance but also on technical accuracy and expression, with scores based on judges' subjective assessments (Jeong and Chun, 2022; Kazemi et al., 2016). To improve in these areas, athletes often repeat the same movements during training (Debien et al., 2020; Jeong and Chun, 2022; Kazemi et al., 2016). This repetitive training, especially during intense periods like the preseason, can increase the risk of overuse injuries. Jeong and Chun (2022) have shown that poomsae athletes experience a higher rate of overuse injuries than those in sparring or demonstration events. This suggests that the specific demands of poomsae training can place significant physical stress. Therefore, when designing preseason training programs, the focus should not be solely on increasing training volume, but also on continuously monitoring athletes’ recovery scores to ensure they remain within an appropriate range.

The findings of this study provide several practical implications. The EWMA ACWR should be prioritized when monitoring preseason TL in Taekwondo athletes. The subjective recovery scale (TQR), although simple, responded sensitively to changes in the EWMA ACWR and may serve as a practical tool for daily TL monitoring. Since baseline recovery levels vary across athletes, EWMA ACWR trends should be interpreted at the group level but applied in combination with individual recovery baselines.

This study also has several limitations. The sample size was relatively small, which may limit the generalizability of the findings. Recovery status was assessed exclusively using a subjective measure (TQR), and no physiological or performance-based indicators were included. Furthermore, we acknowledge that coding rest days as 0 AU in the calculation of s-RPE-based TL indicators represents a methodological limitation of the current study (Impellizzeri et al., 2019). This decision was made to preserve the continuity of the daily workload time series. This approach may influence the estimation of the chronic workload in the denominator of ratio-based indices such as the ACWR (Impellizzeri et al., 2020). Therefore, interpretation of s-RPE-based TL indicators should carefully consider the methodological assumptions related to rest day handling (Bourdon et al., 2017). Future research should examine larger samples to improve generalizability, incorporate diverse physiological and psychological markers (e.g., HRV, sleep quality, cortisol), and further investigate the utility of EWMA ACWR-based TL management for reducing the injury incidence and improving performance. In addition, it should directly compare different rest day handling strategies (e.g., 0 AU vs. missing data) and evaluate how these methodological choices influence the interpretation of TL indicators. Such efforts would contribute to the development of more standardized frameworks for internal TL monitoring.

## Conclusions

The EWMA ACWR was the only TL indicator that consistently predicted recovery status in collegiate TKD athletes. Integrating the EWMA ACWR with simple tools such as the TQR scale may help balance TL and recovery, reduce injury risks, and support performance during the preseason period.
